# Genome Dynamics of Short Oligonucleotides: The Example of Bacterial DNA Uptake Enhancing Sequences

**DOI:** 10.1371/journal.pone.0000741

**Published:** 2007-08-15

**Authors:** Mohammed Bakkali

**Affiliations:** Institute of Genetics, Queen's Medical Center, University of Nottingham, Nottingham, United Kingdom; Pasteur Institute, France

## Abstract

Among the many bacteria naturally competent for transformation by DNA uptake—a phenomenon with significant clinical and financial implications— Pasteurellaceae and Neisseriaceae species preferentially take up DNA containing specific short sequences. The genomic overrepresentation of these DNA uptake enhancing sequences (DUES) causes preferential uptake of conspecific DNA, but the function(s) behind this overrepresentation and its evolution are still a matter for discovery. Here I analyze DUES genome dynamics and evolution and test the validity of the results to other selectively constrained oligonucleotides. I use statistical methods and computer simulations to examine DUESs accumulation in *Haemophilus influenzae* and *Neisseria gonorrhoeae* genomes. I analyze DUESs sequence and nucleotide frequencies, as well as those of all their mismatched forms, and prove the dependence of DUESs genomic overrepresentation on their preferential uptake by quantifying and correlating both characteristics. I then argue that mutation, uptake bias, and weak selection against DUESs in less constrained parts of the genome combined are sufficient enough to cause DUESs accumulation in susceptible parts of the genome with no need for other DUES function. The distribution of overrepresentation values across sequences with different mismatch loads compared to the DUES suggests a gradual yet not linear molecular drive of DNA sequences depending on their similarity to the DUES. Other genomically overrepresented sequences, both pro- and eukaryotic, show similar distribution of frequencies suggesting that the molecular drive reported above applies to other frequent oligonucleotides. Rare oligonucleotides, however, seem to be gradually drawn to genomic underrepresentation, thus, suggesting a molecular drag. To my knowledge this work provides the first clear evidence of the gradual evolution of selectively constrained oligonucleotides, including repeated, palindromic and protein/transcription factor-binding DNAs.

## Introduction

Many bacteria are naturally competent for transformation by spontaneous uptake of DNA from their surrounding environments [1], [2]. Among these, Pasteurellaceae and Neisseriaceae species preferentially take up DNA containing specific short sequences, called uptake signal sequences (USS) and DNA uptake sequences (DUS), respectively [3]–[9]. Both USSs and DUSs are overrepresented in their respective genomes and, while only one DUS (5′-GCCGTCTGAA) has been described [10]–[13], USS was reported in two slightly different versions: *(i)* 5′-AAGTGCGGT in *Haemophilus influenzae* [13]–[15], *Actinobacillus actinomycetemcomitans* [6], [9], [15], *Haemophilus somnus* and *Pasteurella multocida* [15], *Mannheimia succiniciproducens* [16] and, probably *Haemophilus parasuis* [16], [17] and *(ii)* 5′-ACAAGCGGTC in *Mannheimia haemolytica* [16], [18] and *Actinobacillus pleuropneumoniae* [16]. Bacterial competence for natural transformation is best characterized in *Bacillus subtilis* (Bacillaceae), *H. influenzae* (Pasteurellaceae), *Neisseria gonorrhoeae* (Neisseriaceae) and *Streptococcus pneumoniae* (Streptococcaceae) (see [1], [2], [19]–[21]). This work therefore focuses on *H. influenzae* and *N. gonorrhoeae* USS/DUS—for which I will henceforth use the unifying name DNA Uptake Enhancing Sequence (DUES).

Less than 10 DUESs are expected in random sequences of similar length and base composition as *H. influenzae* Rd Kw20 and *N. gonorrhoeae* FA1090 genomes (see Table S1). However, Smith et al. [13], [14] found 1471 DUES in the first genome, and Davidsen et al. [11] counted 1965 in the latter. The search for a function to account for the evolution of these sequences is still inconclusive. Goodman and Scocca [22] suggested a DUES role in transcription termination, and Karlin et al. [23] viewed the significant even spacing of these sequences around the genome as a sign of their role in DNA replication, repair, or compaction. However, any transcription termination activity is difficult to envisage for at least three reasons: *(i)* 65% of *H. influenzae* and 35% of *N. gonorrhoeae* DUESs are located within open reading frames [11], [13], [14], *(ii)* DUESs are not palindromes and only ∼10% occur as inverted repeats (M. Bakkali, unpublished), and *(iii)* there are more DUESs in non-coding regions than expected for an unbiased distribution [15]. Furthermore, no intracellular DUES-binding protein was identified, and DUESs show no orientation bias around the chromosome [13]–[15]—features expected from a sequence that interacts with the replication machinery.

DUESs as bacterial mate recognition systems [24] is the only uptake related function proposed for these sequences. In this case, DUESs could be tags for ‘safe sex’ among bacterial cells seeking recombination trough competence for natural transformation. Given the striking DUES genomic overrepresentation, any preferential uptake of DUES-containing DNA will inevitably result in preferential uptake of conspecific DNA, and DNA from species sharing the same DUES (*i.e.*, closely related species). Hence, DUES-biased system of DNA uptake could evolve given a higher selective advantage of recombination with DNA from conspecifics compared to DNA from unrelated species, and computer simulations seem to confirm this possibility [25]. However, bacteria were suggested to take up DNA mainly as a nutrient rather than for recombination [26], [27]. Increasingly supported by recent findings [28]–[31], this hypothesis raises further questions about the evolution of a DUES-biased DNA uptake system that significantly limits the quantity of DNA available to the competent cells.

*H. influenzae* has ‘only’ 764 singly-mismatched DUESs [13]–[15], even though these can arise from the species' 1471 nine base pairs (bp) long DUESs by any of the 27 possible mutations. Testing this ostensible sequence homogeneity may therefore help understand DUES evolution. High sequence homogeneity is expected in repeats arising by copying, such as transposable elements and telomeres, and Smith et al. [13] suggested that the unexpectedly low frequency of singly-mismatched DUESs relative to the non-mismatched one could be due to a balance between mutation away from the latter and its restoration by preferential uptake. However, DUESs are not known to be transposable, and we have no reason to discard uptake bias in favor of singly-mismatched ones. Both *H. influenzae* and *Neisseria meningitidis* even take up DUES-lacking DNAs, though less efficiently [22], [32]–[34].

Based on sequence comparison of homologous pasteurellacean genes, DUESs were suggested to evolve by gradual accumulation of point mutations in preexisting sequences rather than by insertion/deletion of entire sequences [15]. As incoming DNA can replace homologous chromosomal regions by recombination, and provided there is enough bias towards some mismatched DUESs as well, DNA uptake could generate a drive that gradually imposes the DUES and some of its mismatched forms in the susceptible parts of the genome (*i.e.*, non-coding and unconstrained coding regions). The resulting molecular drive, tested in [35], offers a simpler explanation of DUES accumulation where the perceived sequence homogeneity could be a consequence of stronger uptake bias towards the DUES than its mismatched forms.

Evaluation of these interpretations requires careful analysis of the genomic representation of all the DUES-like sequences as well as the actual bias of DNA uptake and its contribution to DUES evolution. In this work I examine and computer simulate the accumulation of DUESs in bacterial genomes by estimating and analyzing the frequencies of *H. influenzae* and *N. gonorrhoeae* DUESs and all their mutated forms. I subsequently monitor the overrepresentation of DUES nucleotides in sequences with different mismatch loads (*i.e.*, number of mismatched positions compared to the DUES) to detect the genomic footprints of the DNA uptake bias and, thus, infer DUES evolutionary history. I then test whether uptake bias in itself can explain DUES accumulation by correlating the strength of DUES-like sequences, as estimated from the genomic frequencies of their nucleotides, to the uptake bias in their favor. Finally, I analyze the distribution of the genomic frequencies of several pro- and eukaryotic sequences to test whether the DUES mode of evolution could be extrapolated to other selectively constrained oligonucleotides. DUES offers a precious system for the study of such sequences—especially protein/transcription factor-binding—as it is selectively bound by the extracellular receptor for DNA uptake, and the uptake bias could be analogous to any other function/selective force.

## Results

### Sequence frequencies and overrepresentation

This analysis aims at answering two questions: (i) Do DUESs evolve by gradual accumulation of mutations? If so, then (ii) what is the minimum number of matches a sequence needs to share with the DUES for its uptake to be significantly preferential?

For a gradual evolution of DUES to take place, a degree of DNA uptake bias towards some of its mutated forms is also required. In such conditions, and assuming that there is no interference from other evolutionary forces than mutation and DNA uptake bias, over evolutionary time the latter should leave a trace in the genome in form of significant overrepresentation of the DUES and its mutated yet preferentially taken up forms. Furthermore, the magnitude of overrepresentation should inversely correlate to the mismatch load of the sequence compared to the DUES. However, no significant overrepresentation is expected for sequences not preferentially taken up and, if any, it does not have to correlate with the mismatch load.

**Figure 1:**
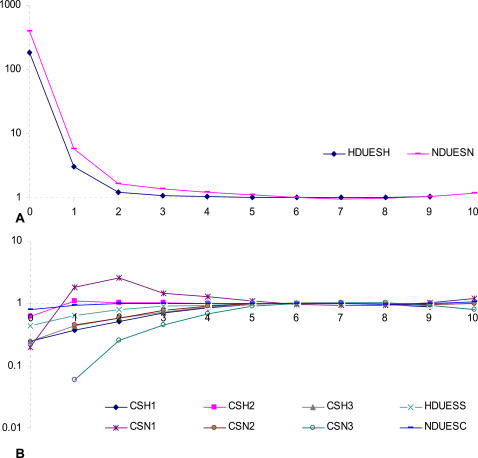
Ratio of the observed to the expected number of sequences across mismatch loads. X-axis: Mismatch load. A: DUESs. B: Control sequences. HDUESH and HDUESS refer to *H. influenzae* DUES in *H. influenzae* and *S. pyogenes* genomes, respectively. NDUESN and NDUESC refer to *N. gonorrhoeae* DUES in *N. gonorrhoeae* and *C. tepidum* genomes. CSH1, 2, and 3 and CSN1, 2, and 3 refer to the three control sequences in *H. influenzae* and *N. gonorrhoeae* (see Material and methods).

Only DUESs in *H. influenzae* and *N. gonorrhoeae* genomes show significant overrepresentation that consistently decreases with the increase in mismatch load until underrepresentation at mismatch load 6 (Figure 1, Table 1, and Table S1). Significant overrepresentation then reappears at the last two sequence categories where it increases with the mismatch load. As expected from non-accumulating DNAs, the control tests show neither overrepresentation of the sequences analyzed, nor any consistent trend in sequence frequencies with respect to the mismatch load. Thus, if sequence overrepresentation is indicative of DNA uptake, these results suggest that DUESs gradually evolve in the genome by accumulation of point mutations.

**Table 1:** Sequence representation (equation 2) of DUESs, control sequences, and all their mismatched forms.

Mismatch load	*H. influenzae*				*S. pyogenes*	*N. gonorrhoeae*				*C. tepidum*
	HDUESH	CSH1	CSH2	CSH3	HDUESS	NDUESN	CSN1	CSN2	CSN3	NDUESC
0	182.875	−0.750	−0.375	−0.750	−0.556	392	−0.800	−1.000	−1.000	−0.200
1	2.008	−0.621	0.099	−0.553	−0.360	4.776	0.828	−0.545	−0.940	−0.048
2	0.220	−0.479	0.029	−0.418	−0.195	0.652	1.667	−0.410	−0.746	0.010
3	0.069	−0.281	0.030	−0.267	−0.095	0.344	0.503	−0.214	−0.548	0.018
4	0.024	−0.133	0.018	−0.123	−0.044	0.190	0.312	−0.080	−0.311	0.012
5	0.001	−0.022	0.001	−0.023	−0.003	0.085	0.098	−0.002	−0.096	0.016
6	−0.012	0.034	−0.003	0.040	0.012	−0.011	−0.014	0.022	0.023	0.010
7	−0.010	0.044	−0.004	0.035	0.010	−0.051	−0.065	0.014	0.059	−0.003
8	0.004	−0.008	0.001	−0.015	−0.002	−0.037	−0.051	−0.012	0.046	−0.017
9	0.032	−0.124	0.010	−0.096	−0.035	0.027	0.038	−0.010	−0.043	−0.004
10						0.176	0.223	0.014	−0.183	0.065

Sequence names as in Figure 1. Significant values, as by χ^2^ test, are underlined (see Table S1).

The distribution of the overrepresentation values is strikingly similar between *H. influenzae* and *N. gonorrhoeae*, suggesting similar DUES genome dynamics and evolution. Nonetheless, overall, *N. gonorrhoeae* values are noticeably higher than those of *H. influenzae*, possibly due to more efficient/frequent DNA uptake. Two major drops in overrepresentation values can easily be identified in both species: The first between mismatch loads 0 and 1 (92 fold in *H. influenzae* and 82 in *H. gonorrhoeae*), and the second between loads 1 and 2 (9 fold in *H. influenzae* and 7 in *N. gonorrhoeae*). This reflects a non-linear relation between the mismatch load and DNA uptake, possibly due to high specificity of the receptor binding the DUES. Coinciding with the base length difference between *N. gonorrhoeae* and *H. influenzae* DUESs, significant overrepresentation values reach one mismatch further in the first species than in the latter. This highlights that for DNA uptake what matters is the number of matches to the DUES not the mismatches.

At four matches (*i.e.*, mismatch load 5 in *H. influenzae*, and 6 in *N. gonorrhoeae*), the results are ambiguous with non-significant overrepresentation in *H. influenzae*, but significant underrepresentation in *N. gonorrhoeae*. This may be due to: *(i)* Non-preferential uptake, *(ii)* insufficient uptake for balancing sequence loss by mutational decay, *(iii)* insufficient drive from higher mismatch loads to balance the drive towards lower ones, or *(iv)* functional constraint selecting against some sequences at this mismatch loads. Sequences with 3 and 2 matches to the DUES are significantly underrepresented, thus, probably not preferentially taken up. They could possibly comprise the pool from which the drive imposed by the DNA uptake bias receives new sequences after mutation. Overrepresentation of sequences with 0 and 1 match to the DUES shows no negative correlation with the mismatch load and is clearly due to uptake independent factor(s) (*e.g.*, functional and mutational constraints or codon bias).

### Computer simulations

**Figure 2:**
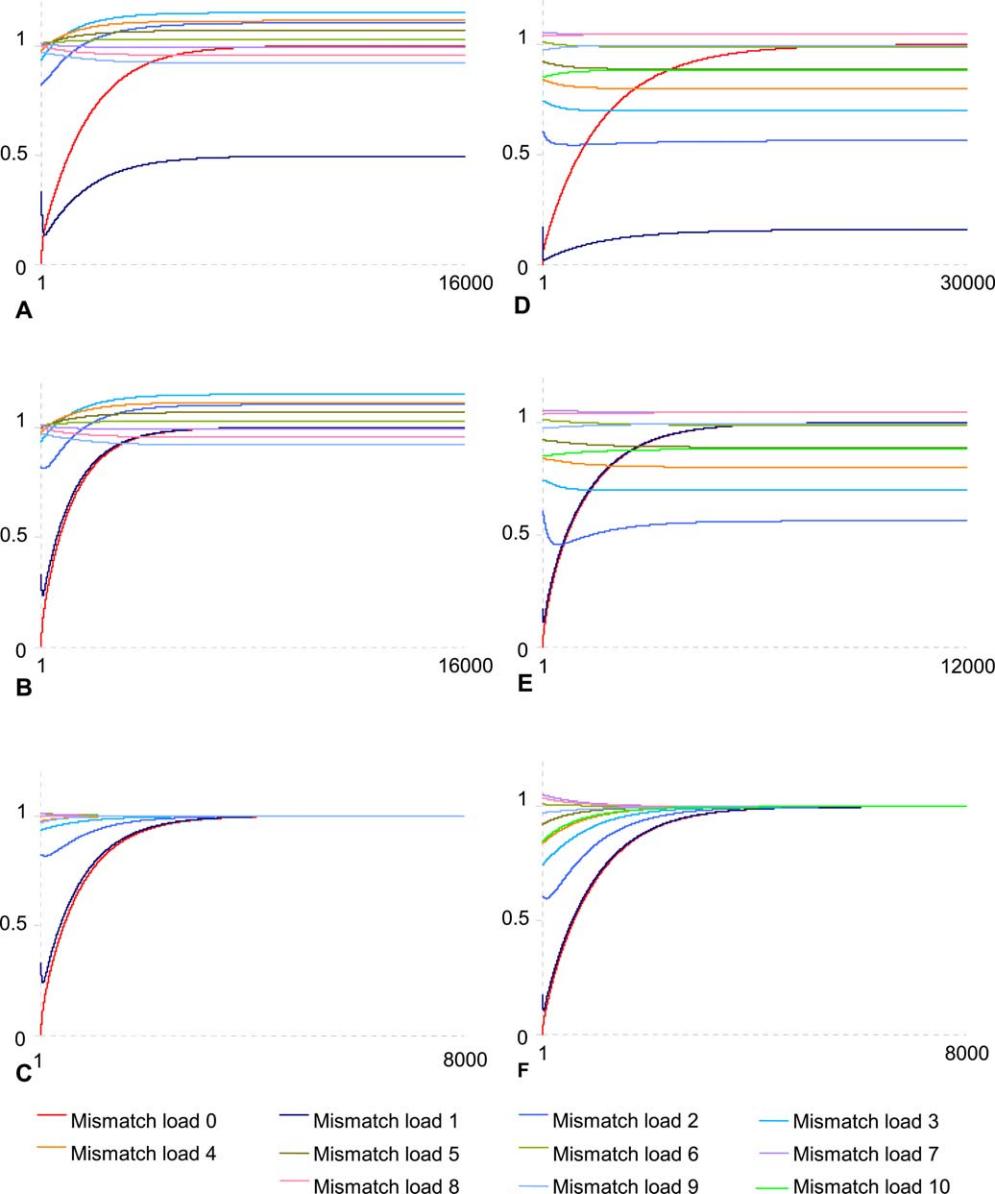
Computer simulation of the accumulation of *H. influenzae* and *N. gonorrhoeae* DUESs. X-axis: Cycle (*i.e.*, generation). Y-axis: Ratio of the number of sequences after each simulation cycle to the observed (*i.e.*, real) number. A and D: Genomes of *H. influenzae* (A) and *N. gonorrhoeae* (D) evolving with 104.4 and 477.5 uptake bias towards their respective DUES. B and E: The same genomes evolving with an additional 1.02 and 5.284 uptake bias towards the singly-mismatched DUESs. C and F: The same genomes evolving with the uptake bias combinations (156.4; 2.016; 0.352; 0.295; 0.340; 0.395; 0.449; 0.505; 0.563; 0.623) and (499.2; 5.567; 0.857; 0.493; 0.306; 0.175; 0.056; 0; 0; 0.051; 0.184) towards sequences with 0 to 9 and 0 to 10 mismatches to the respective DUES—semicolons separate uptake biases for different mismatch loads.

Overrepresentation of mismatched sequences suggests that they are preferentially taken up. Still, it could also be due to mutational decay of sequences with fewer mismatches that are preferentially taken up. DUESs are so overrepresented that singly-mismatched sequences could reach overrepresentation only by DUES mutational decay—as suggested in [13]. It is also possible that the two major drops in sequence overrepresentation reported above reflect uptake bias only towards sequences with less than 2 mismatches to the DUES. I therefore simulated the evolution of sequence frequencies in a population of 10^18^ genomes with the same distribution of sequence frequencies as those expected for randomized *H. influenzae* and *N. gonorrhoeae* genomes (see Table S1) evolving at 10^−2^ mutations per base per generation. Mutations alone are not able to drive neither *H. influenzae* nor *N. gonorrhoeae* DUES to their respective observed overrepresentation levels (data not shown), and an uptake bias of 104.4 and 477.5 respectively is needed (Figure 2A;D). However, the results do not support the possibility of mutational decay. By themselves, these uptake biases can neither drive the singly-mismatched sequences to their observed overrepresentation levels, nor give similar distributions of sequence frequencies as the observed ones (Figure 2A;D). An additional 1.02 and 5.284 uptake bias in favor of singly-mismatched *H. influenzae* and *N. gonorrhoeae* DUESs was needed to attain their observed frequencies, yet it did not give similar overall sequence frequency distribution as the observed one (Figure 2B;E). The same was true for additional uptake biases towards other mismatch loads (data not shown). An approximation to the observed distributions of sequence frequencies with an error margin of less than 0.01% was obtained only with the distributions of uptake biases shown in Figure 2C;F. This distribution agrees with my interpretation of the sequence overrepresentation results, as it supports uptake bias towards mismatched sequences and negative correlation between the mismatch load and the uptake bias. The exception seems to be *H. influenzae* sequences at mismatch loads 4 to 9, where uptake bias value increases with the mismatch load, which may reflect the involvement of other selective forces than uptake. The values of uptake bias used in the simulations certainly include the other selective forces, such as functional constraints, that led to the observed distributions of sequence frequencies in the real genomes.

To reach the currently observed genomic distributions of sequence frequencies, simulations needed higher uptake bias values and more cycles (*i.e.*, generations) for *N. gonorrhoeae* than for *H. influenzae* (Figure 2C;F). This may be due to: *(i)* Differences in the ancestral organization of the real genomes which, obviously, was not random as assumed in the simulations, *(ii)* more frequent uptake of DNA by the almost permanently competent *N. gonorrhoeae* [36], [37] than by the occasionally competent *H. influenzae* [4], [38], [39], or *(iii)* earlier evolution of the DUES-biased DNA uptake in *N. gonorrhoeae* than in *H. influenzae*.

### Sequence logos and evolution of DUES nucleotides

The results reported above clearly indicate gradual evolution of DUESs by accumulation of point mutations. However, this is only certain if we see a gradual increase in the overrepresentation of the actual DUES nucleotides; as nucleotide overrepresentation at any particular position of the DUES may not show any trend with regards to the mismatch level.

**Figure 3:**
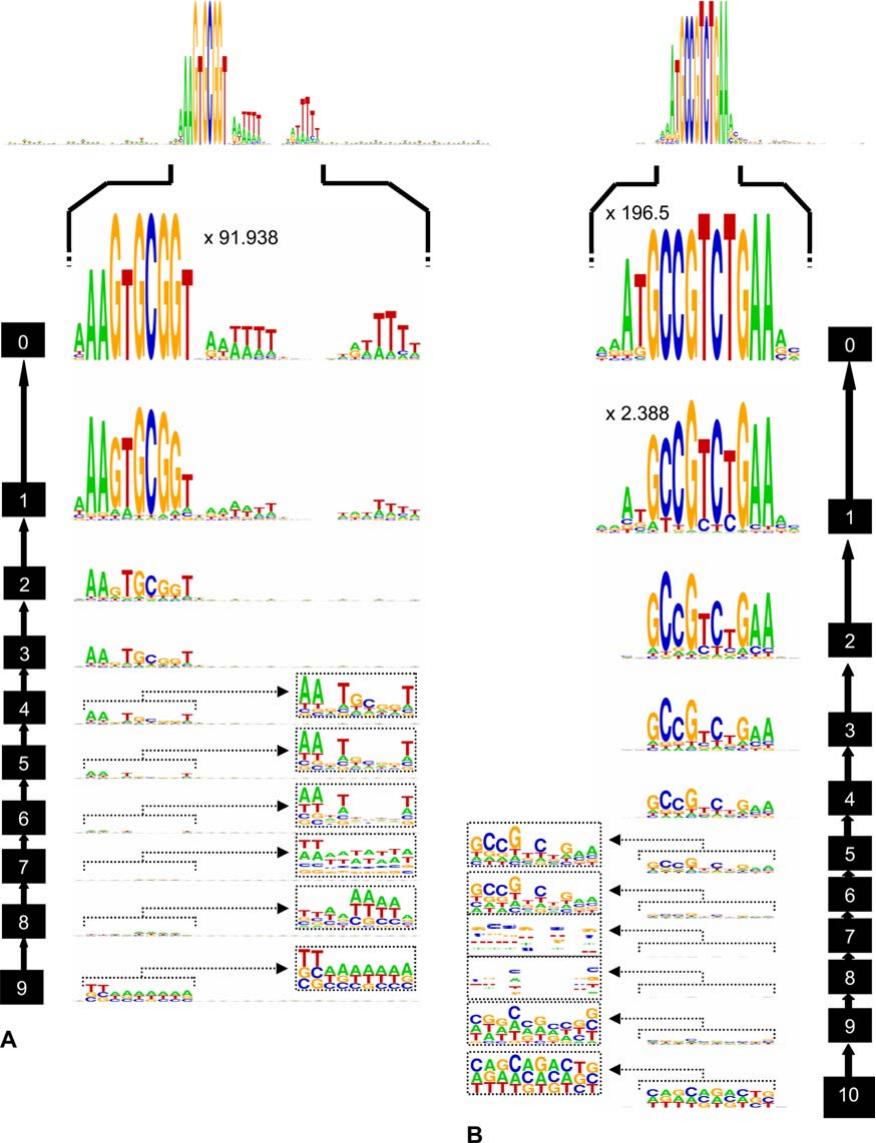
DNA sequence logos showing the accumulation of *H. influenzae* and *N. gonorrhoeae* DUES nucleotides. The heights of the characters reflect the conservation of the nucleotides ([equation 3 in 66]) as well as the genomic representation of sequences at the mismatch load (equation 2). The number to the side of each logo is the mismatch load analyzed. The height of the logo should be multiplied by the number at the top, if applicable.

So far I focused solely on the 9 and 10 bp H. influenzae and N. gonorrhoeae DUESs. However, for H. influenzae, this sequence is only the core of a larger (29 bp) DUES consensus containing two additional less conserved regions [14], [16]. Conversely, there is no report of less conserved DUES regions in N. gonorrhoeae [7]. DNA sequence logos were therefore generated for 100 pb sequences, containing the DUES cores or one of their mismatched forms, in order to include the less conserved regions and look for possible additional ones. H. influenzae DUES consensus is 5′-aAAGTGCGGTnrwttttnnnnnnrwtttw, where r = A or G and w = A or T (Figure 3A), which is similar to the consensus reported in [14]. N. gonorrhoeae DUES also seems to show less conserved nucleotides (Figure 3B), especially an adenine and a thymine at the 5′ side. Its consensus sequence thus being 5′-mdatGCCGTCTGAAvv, where d = A, G or T, m = A or C and v = A, G or C.

The logos support previous interpretations, as they show clear gradual accumulation of DUES nucleotides starting from the third match, with some nucleotides gaining overrepresentation earlier/faster than others, probably due to their importance for uptake. This, together with the results of the simulations, solves the ambiguity regarding sequences with 4 matches and suggests a certain bias in their uptake, though not sufficient to drive them to significant overrepresentation. Coinciding with the biases in genomic base composition, DUES Ws emerge first in H. influenzae whilst, in N. gonorrhoeae, the whole DUES core emerges at the same time with a slightly higher overrepresentation of Ss than Ws. Unlike the core, DUES nucleotides of the less conserved regions emerge only after mismatch load 2 and, in H. influenzae positions 14 and 15, there is a switch in the nature of the most overrepresented nucleotide; probably because of similarity in their overrepresentation. In neither of the species does overrepresentation seem to depend on uptake at less than 3 matches, since the logos do not show resemblance to the respective DUES cores.

### Dependence of the uptake bias on the strength of the sequence

Dependence of sequence overrepresentation on DNA uptake is the key to interpreting the results described above. I therefore used the 28 DNA fragments tested for uptake efficiency by competent *H. influenzae* in [40]—which are the best experimental data set available on DNA uptake—to test the dependence of their uptake on the strength (equation 19) of their 29 bp DUES-like sequences. The small differences in the naming and sizes of some of the DNA fragments between what is reported in [40] and the sequences available at the database are insignificant and will not affect the intended analyses. 15 of these fragments were classified by Goodgal and Mitchell [40] as uptake fragments and the remaining 13 as non-uptake. As Table 2 shows, the uptake fragments are, overall, larger than the non-uptake ones, and the average strength of their strongest 29 bp sequences is higher, whilst the average mismatch load of these sequences is lower. The mean mismatch load of all the 29 bp sequences of every uptake and non-uptake fragment is similar, whereas their mean strength is lower in the uptake than the non-uptake fragments. In principle, this may be sufficient to infer dependence of the uptake efficiency on the strength of the strongest sequence of a DNA fragment. However, DNAs were named by Goodgal and Mitchell [40] prior to quantification of their uptake, and their classification into uptake and non-uptake fragments does not reflect the clear division between fragments taken up at more than 60 molecules per cell and those taken up at 30 or less. Three fragments originally classified as non-uptake are better seen as uptake fragments (14nu, 27nu and 58nu), and six *vice versa* (159u, 201u, 205u, 30u, 62u and 71u). After this reclassification, the overall uptake fragments (*i.e.*, all their 29 bp sequences), on average, show less mismatches, but less strength, than the non-uptake ones, whereas their strongest 29 bp sequences show less mismatches and more strength.

**Table 2:** Characteristics of the DNA fragments tested for uptake in [40].

Fragment	Characteristic	Number of fragments	Mean	Minimum	Maximum	Standard Deviation
Uptake	Mismatch load of the strongest sequence	15 (12)	1.467 (0.167)	0 (0)	5 (1)	1.807 (0.389)
	Mean mismatch load of all the 29 bp sequences		6.817 (6.820)	6.763 (6.774)	6.89 (6.89)	.029 (0.033)
	Strength of the strongest sequence ×10^6^		136014.044 (157773.523)	93438.933 (145161.802)	164318.553 (165301.003)	27130.732 (5570.456)
	Mean strength of all the 29 bp sequences ×10^6^		31734.307 (31549.857)	26186.814 (26186.814)	35824.358 (34947.958)	2187.048 (2486.952)
	Length of the DNA fragment		177.133 (170.833)	94 (85)	295 (295)	55.631 (67.261)
	Uptake of the DNA fragment		51.227 (72.833)	6.4 (67)	80 (80)	28.125 (4.282)
Non-uptake	Mismatch load of the strongest sequence	13 (16)	2.385 (3.188)	0 (2)	4 (5)	1.325 (0.75)
	Mean mismatch load of all the 29 bp sequences		6.806 (6.806)	6.72 (6.72)	6.88 (6.88)	0.039 (0.034)
	Strength of the strongest sequence ×10^6^		116059.201 (103481.125)	75958.096 (75958.096)	165301.003 (116996.024)	27537.782 (10406.055)
	Mean strength of all the 29 bp sequences ×10^6^		32571.148 (32552.578)	26924.022 (26924.022)	39076.779 (39076.779)	2941.195 (2591.2731)
	Length of the DNA fragment		132.154 (145.313)	50 (50)	311 (311)	73.043 (66.938)
	Uptake of the DNA fragment		24.946 (13.669)	6 (6)	79 (30)	28.146 (8.892)

Between parentheses are the results after reclassification of the uptake and non-uptake fragments (see Results).

Regression analysis to detect dependence of the number of DNA molecules taken up by competent *H. influenzae* on the strength and mismatch load of their sequences shows very significant values for the strongest 29 bp sequence in each of the 28 DNA fragments (Table 3). This suggests that the efficiency of DNA uptake depends on the strength of the best region of the DNA fragment. However, the results are also significant for the second 29 bp sequences in strength, implying that the preferential uptake might not always target the strongest sequence in a DNA fragment. Nevertheless, weaker sequences show no significant results, and the significance of the regression is much higher for the strongest sequence. The significant results of the second sequences in strength could, thus, be due to similarities with the strongest ones in some DNA fragments. In fact, 11 DNA fragments show less than 5% difference in strength between their strongest and the following sequence (fragments 8u2, 30u, 37nu2, 39nu, 45nu, 48u, 51nu, 71u, 159u, 201u, 205u). At 10% cut-off, the number of these fragments already reaches half the sample size (due to addition of fragments 43u, 48nu, 62nu).

**Table 3:** Dependence of the uptake efficiency of the DNA fragments in [40] on the strength and mismatch load of their 29 bp sequences.

29 bp sequence	Characteristic	*BETA*	*t_26_*	*p-level*
Strongest	Strength	0.894	10.171	**<10^−9^**
	Mismatch load	−0.867	−8.901	**<10^−8^**
Second	Strength	0.520	3.103	**0.005**
	Mismatch load	−0.418	−2.349	**0.027**
Third	Strength	0.295	1.574	0.128
	Mismatch load	−0.239	−1.253	0.221
Fourth	Strength	0.288	1.536	0.137
	Mismatch load	0.055	0.283	0.779
Fifth	Strength	0.260	1.374	0.181
	Mismatch load	−0.311	−1.668	0.107

Only the data from the top five strongest 29 bp sequences are shown here.

Even though uptake DNA fragments on average are larger than the non-uptake ones (Table 2), DNA uptake efficiency does not depend on the length of the DNA fragment taken up (Table 4). However, had I tested more DNA fragments, such effect could have been detected as, in theory, the larger a DNA fragment is, the more likely it is to have a strong DUES. Furthermore, neither the mean mismatch load of all the 29 bp sequences of a DNA fragment, their mean strength, the position of the strongest one in the fragment, nor its orientation have any significant effect on DNA uptake (Table 4). Randomizing the 29 positions of the strongest sequence in each DNA fragment abolishes the significant dependence of the original uptake values on the strength of these sequences (*e.g.*, *Beta* = 0.271, *t_26_* = 1.433 and *p-level* = 0.164).

**Table 4:** Lack of dependence of uptake efficiency on the length of the DNA fragment, its average mismatch load, strength, and the position and orientation of its strongest DUES.

Characteristic of the DNA fragment	*BETA*	*t_26_*	*p-level*
Length	0.159	0.823	0.418
Mean mismatch load	0.258	1.361	0.185
Mean strength	−0.251	−1.320	0.198
Position of the strongest 29 pb sequence	0.013	0.066	0.948
Orientation of the strongest 29 pb sequence	0.112	0.577	0.569

Ultimately, the results suggest that the efficiency of DNA uptake significantly depends on the strength of one sequence only—the strongest—which is the most likely to be bound and taken up independently of its position or orientation in the DNA fragment. Since the strength of sequences was estimated based on the genomic overrepresentation (conservation) of their nucleotides (see equation 19), these results suggest that DNA uptake bias alone can explain the genomic overrepresentation of DUESs and some of their mismatched forms.

### Experimental verification of the theoretical results

For experimental confirmation of the abovementioned results, uptake was measured for 222 bp DNA fragments generated *in vitro* to contain the ‘best’ DUES consensus, consensuses mutated to the least frequent nucleotide at positions 2–11, 13 and 14, 20, or 25 and 26, or the worst 29 bp sequence. Uptake experiments were replicated several times and for different amounts of DNA (10, 20, 30 and 40 ηg). The data show considerable variance between experiments, and a nested ANCOVA was performed on the uptake values and their standard deviations to detect possible dependence of uptake on the availability of DNA and the strength of the DUES, as well as to estimate the magnitude of the noise (*i.e.*, error) emanating from the variation between experiments. The results (Table 5) suggest that the amount of DNA taken up strongly depends on the nature of the DUES (*i.e.*, stronger DUESs are taken up more efficiently), and on the amount of DNA given to the cells (*i.e.*, at non-saturating concentrations, as in these experiments, more DNA in the medium increases the possibility for cells to ‘find’ it, bind it, and take it up). Nonetheless, the percentage of DNA taken up depends only on the nature of the DUES. The variability between experiments, however, could be due to differences in the uptake/transformation efficiency (*i.e.*, competence state) of the cells used in each experiment and/or the use of different DNA concentrations and/or DUESs in different experiments. This may be the reason why the variation in the amount of DNA taken up is only explained by variation in the strength of the DUES and the amount of DNA given to the cells, whereas the variation in the percentage of DNA taken up solely depends on the nature of the DUES tested (*i.e.*, position mutated).

**Table 5:** Effect of the DNA concentration in the medium, the strength of the DUES, and the experiment on DNA uptake efficiency by competent *H. influenzae*.

Dependant variable		Effect (*df*)	*MS Effect*	*MS Error*	*F*	*p*
Nanograms of DNA taken up	Mean	DUES (14)*	22.474	1.043	21.554	**0**
		DNA (1)**	30.908	2.254	13.712	**0.003**
		Experiment (14)***	2.128	0.902	2.359	**0.011**
	Standard deviation	DUES (14)*	0.183	0.046	4.004	<**0.0001**
		DNA (1)**	0.340	0.059	5.730	**0.034**
		Experiment (14)***	0.058	0.044	1.316	0.224
Percentage of DNA taken up	Mean	DUES (14)*	347.15	12.754	27.219	**0**
		DNA (1)**	119.129	29.2	4.08	0.065
		Experiment (14)***	27.492	10.844	2.535	**0.006**
	Standard deviation	DUES (14)*	2.735	0.829	3.298	**0.0004**
		DNA (1)**	1.074	1.178	0.912	0.358
		Experiment (14)***	1.141	0.789	1.447	0.159

The test used is a nested ANCOVA.

* Fixed effect.

** Covariate effect.

*** Random effect.

**Figure 4:**
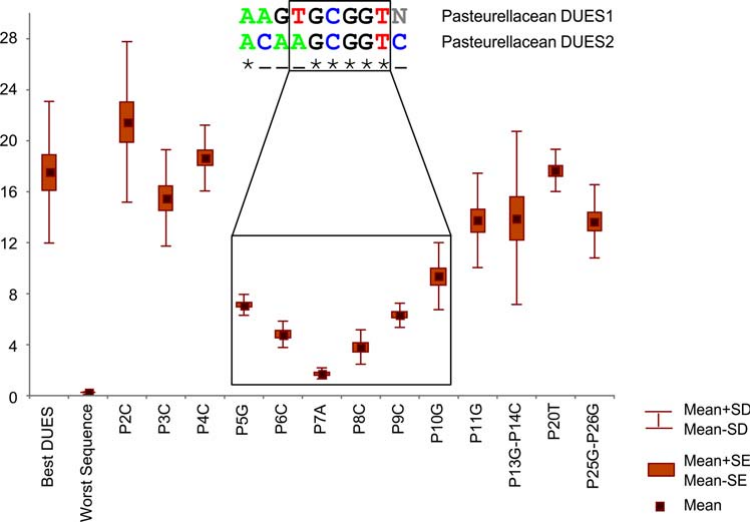
Relative uptake efficiency of *H. influenzae* DUES and some of its mismatched forms. X-axis: Sequence tested. Best DUES: *H. influenzae* best DUES consensus. Worst Sequence: The 29 bp sequence of the least frequent nucleotides. PxN: Best DUES mutated at position x (Px) towards the least frequent nucleotide (N). Y-axis: Percentage of DNA taken up. SD: Standard deviation of the mean, SE: Standard error. Note that the critical part of the DUES largely coincides with the evolutionarily conserved positions of the two pasteurellacean DUESs currently known.

Overall, the experimental findings agree with those of the theoretical analysis reported above, and mutations of theoretically important DUES positions also result in less DNA uptake (Figure 4). The little effect on uptake after mutating the second, third and fourth positions of the DUES consensus, however, do not agree with their conservation results. Still, this result could reflect the evolutionary variability of nucleotides at these positions as they show differences between the two pasteurellacean DUESs (Figure 4). The experiment confirms that, even within the DUES core, the importance of the different positions for DNA uptake varies, with the evolutionarily conserved ones being the most important. Indeed, Karlin et al. [23] reported a biased distribution of singly mismatched DUESs in *H. influenzae* genome.

### The findings on DUES could be generalized to other oligonucleotides

**Figure 5:**
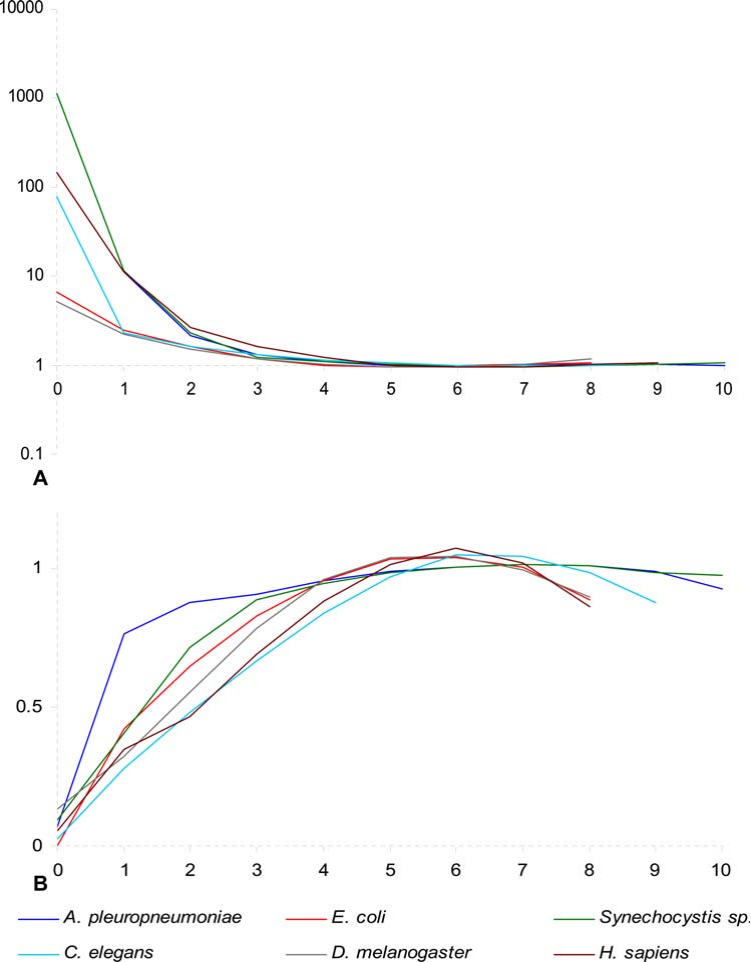
Ratio of the observed to the expected number of several pro- and eukaryotic oligonucleotides in their respective genome/chromosome. X-axis: Mismatch load. A: Sequences genomically overrepresented. B: Sequences genomically underrepresented. The sequences are the same as in Tables 6 and 7.

In this work I took advantage of the significant amount of experimental data on DUES mediated DNA uptake and the convenience of working with bacterial genomes, and used *H. influenzae* and *N. gonorrhoeae* DUESs as examples of selectively driven—protein-binding—oligonucleotides. As Figure 5A;B and Tables 6 and 7 show, the gradual mode of evolution suggested by the results on these DUESs seems to be a common feature of other selectively constrained oligonucleotides, including the other pasteurellacean DUES as well as several other pro- and eukaryotic short repeated, palindromic and protein-binding DNAs. Sequences showing genomic overrepresentation seem to gradually evolve in the same way as the DUES (*i.e.*, by molecular drive), whilst those underrepresented seem to evolve in an inversed fashion (*i.e.*, by molecular drag). The differences in over- or underrepresentation levels between sequences and mismatch loads reflect the differences in strength and specificity of the selective force(s) directing the evolution of these sequences.

**Table 6:** Genomic frequencies of various prokaryotic oligonucleotides and their mismatched forms showing signs of gradual drive or drag towards genomic over- or underrepresentation.

Mismatch load	*Actinobacillus pleuropneumoniae* str. L20 Complete genome (∼2.28 Mb, 41.3% G+C) Accession no. NC_009053				*Escherichia coli str. K12* Complete genome (∼4.64 Mb, 50.79% G+C) Accession no. NC_000913				*Synechocystis* sp. PCC 6803 Complete genome (∼3.57 Mb, 47.72% G+C) Accession no. NC_000911			
	5′-ACAAGCGGTC (DUES [16])				5′-GCTGGTGG (Chi sequence; a recombination hotspot [69])				5′-GGCGATCGCC (Palindrome frequent in cyanobacteria [70]. Contains the recognition site of a DNA methyltransferase [71] and of the endonuclease SgfI)			
	*Seq_exp_*	*Seq_obs_*	*Rep_seq_*	χ*^2^*	*Seq_exp_*	*Seq_obs_*	*Rep_seq_*	Χ*^2^*	*Seq_exp_*	*Seq_obs_*	*Rep_seq_*	Χ*^2^*
0	3	438	145.000	63075.042	151	1008	5.675	4863.980	5	5648	1128.600	6368694.256
1	86	947	10.012	8620.175	3579	8855	1.474	7780.642	160	1830	10.438	17431.015
2	1252	2635	1.105	1528.127	37198	60106	0.616	14164.433	2244	5212	1.323	3926.823
3	10796	14278	0.323	1125.710	220892	259638	0.175	6962.048	18646	22668	0.216	869.827
4	60767	66466	0.094	541.714	819776	822254	0.003	8.216	101666	112708	0.109	1216.584
5	233206	233417	0.001	0.201	1947014	1849597	−0.050	−3579.891	380012	391692	0.031	379.155
6	617875	602744	−0.024	−312.299	2890031	2823959	−0.023	−827.290	986147	966932	−0.019	−314.473
7	1115838	1098244	−0.016	−187.249	2451193	2490658	0.016	863.497	1754338	1692644	−0.035	−1463.755
8	1314386	1323863	0.007	96.098	909516	963275	0.059	3522.838	2047556	2036808	−0.005	−33.765
9	911850	923384	0.013	182.470					1415774	1451248	0.025	1108.418
10	282906	282548	−0.001	−0.423					440394	459550	0.043	887.952

Mismatch load	5′-GAATATAATA (Unknown effect. Contains the -10 sequence consensus 5′-TATAAT)				5′-GCCTAGGC (Palindrome of unknown effect. CCTAGG is the recognition site of many restriction endonucleases including: AvrII, BlnI, BspA2I and XmaJI)				5′-TATATAATTA (Unknown effect. Contains the -10 sequence consensus 5′-TATAAT and two palindromic parts: TATATA And TAATTA)			
	*Seq_exp_*	*Seq_obs_*	*Rep_seq_*	χ*^2^*	*Seq_exp_*	*Seq_obs_*	*Rep_seq_*	Χ*^2^*	*Seq_exp_*	*Seq_obs_*	*Rep_seq_*	χ*^2^*
0	15	1	−0.933	−13.067	151	0	−1.000	−150.998	11	1	−0.909	−9.091
1	387	296	−0.235	−21.396	3579	1512	−0.578	−1193.306	301	123	−0.591	−105.258
2	4431	3886	−0.123	−66.968	37198	24062	−0.353	−4620.141	3825	2726	−0.287	−315.596
3	29955	27167	−0.093	−257.767	220892	182658	−0.173	−6456.511	28818	25482	−0.116	−384.615
4	132571	126836	−0.043	−240.648	819776	782972	−0.045	−1492.204	142498	134571	−0.056	−431.999
5	401381	397544	−0.010	−33.130	1947014	2009660	0.032	2550.896	483165	475749	−0.015	−105.574
6	842140	844831	0.003	10.552	2890031	3005796	0.040	6734.650	1137673	1143992	0.006	41.742
7	1209253	1225989	0.014	315.493	2451193	2464552	0.005	98.943	1836890	1864714	0.015	567.254
8	1137496	1149841	0.011	178.650	909516	808138	−0.111	−10072.043	1946338	1961050	0.008	152.824
9	633039	625898	−0.011	−67.532					1222108	1202330	−0.016	−254.056
10	158296	146675	−0.073	−822.376					345313	336202	−0.026	−228.187

*Seq_exp_*: Expected number of sequences, *Seq_0bs_*: Observed number of sequences, χ*^2^*: Goodness of fit Chi-squared, and *Rep_seq_*: Sequence representation (equation 2). Significant values are underlined. Positive values mean overrepresentation, whereas negatives mean underrepresentation (equation 3).

**Table 7:** Genomic frequencies of various eukaryotic oligonucleotides and their mismatched forms showing signs of gradual drive or drag towards genomic over- or underrepresentation.

Mismatch load	*Caenorhabditis elegans* Chromosome V (∼20.9 Mb, 35.43% G+C). Accession no. NC_003283.6				*Drosophila melanogaster* Chromosome 3R (27.9 Mb, 42.91%G+C). Accession no. NT_033777.2				*Homo sapiens* Partial chromosome 21 (∼28.6 Mb, 39.12% G+C). Build 36.2, contig Accession no. NT_011512.10			
	5′-GCAGCGCCC (Unknown function)				5′-AAATATTT (Palindrome of unknown function)				5′-CCAGCCTGG (Unknown function [62], highly conserved across mammalian and other vertebrates' Alu-like repeats [72])			
	*Seq_exp_*	*Seq_obs_*	*Rep_seq_*	χ*^2^*	*Seq_exp_*	*Seq_obs_*	*Rep_seq_*	χ*^2^*	*Seq_exp_*	*Seq_obs_*	*Rep_seq_*	χ*^2^*
0	13	1022	77.615	78313.947	2460	12562	4.107	41485.732	58	8537	146.190	1239543.342
1	514	1161	1.259	814.424	49266	107792	1.188	69587.929	1938	21892	10.296	205456.965
2	8936	14271	0.597	3185.800	431635	658690	0.526	120369.746	28579	75764	1.651	77943.122
3	90125	119514	0.326	9604.193	2160971	2537278	0.174	68168.806	244451	399058	0.632	98203.132
4	581112	667354	0.148	12979.326	6761788	6886084	0.018	2599.804	1335657	1619802	0.213	61892.796
5	2481719	2590153	0.044	5036.568	13541106	12684254	−0.063	−36850.113	4831548	4842194	0.002	25.621
6	7011409	6891951	−0.017	−1625.544	16948322	15939888	−0.060	−33834.194	11562803	10915084	−0.056	−27097.660
7	12616714	12241133	−0.030	−6353.284	12121636	12466476	0.028	12531.974	17640150	16924950	−0.041	−16078.306
8	13093968	13109859	0.001	28.071	3792921	4517082	0.191	148341.405	15554578	15967840	0.027	15077.269
9	5954282	6202374	0.042	12052.253					6035096	6459737	0.070	33400.451

Mismatch load	5′-CATTGGACG (Unknown effect)				5′-CCTATAGG (Palindrome of unknown effect)				5′-GCGATCGC (Palindrome of unknown effect. Recognition site of the restriction endonuclease SgfI)			
	*Seq_exp_*	*Seq_obs_*	*Rep_seq_*	χ*^2^*	*Seq_exp_*	*Seq_obs_*	*Rep_seq_*	χ*^2^*	*Seq_exp_*	*Seq_obs_*	*Rep_seq_*	χ*^2^*
0	79	2	−0.975	−75.050	785	104	−0.868	−590.770	297	16	−0.946	−265.861
1	2507	706	−0.718	−1293.740	19359	6224	−0.678	−8908.950	8686	3038	−0.650	−3672.008
2	34655	16673	−0.519	−9322.876	207778	115132	−0.446	−41155.497	110391	51402	−0.534	−31460.696
3	274561	182469	−0.335	−30685.035	1267802	994176	−0.216	−57683.182	795769	549984	−0.309	−74843.963
4	1372461	1148319	−0.163	−35363.994	4809895	4614934	−0.041	−7157.129	3555936	3130212	−0.120	−47592.167
5	4484837	4339653	−0.032	−4135.636	11618339	12078072	0.040	22974.115	10077472	10210168	0.013	2120.679
6	9574735	10039015	0.048	29193.983	17449545	18193432	0.043	46137.890	17671185	18958974	0.073	135764.977
7	12876141	13457633	0.045	37935.219	14899055	14833324	−0.004	−184.380	17512517	17863884	0.020	10157.788
8	9900467	9739508	−0.016	−1805.644	5537548	4974708	−0.102	−50905.998	7502606	6467180	−0.138	−121340.368
9	3318349	2914814	−0.122	−44845.367								

Abbreviations as in Table 6. Significant values are underlined. Positive values mean overrepresentation, whereas negatives mean underrepresentation (equation 3).

## Discussion

The current names of pasteurellacean uptake signal sequences (USS) and neisseriacean DNA uptake sequences (DUS) could be misleading, as they do not accurately describe the effect of these sequences on DNA uptake and imply that they represent different genetic elements. Both sequences show similar genomic distributions in the corresponding genomes [9], [10], [12]–[14], and have the same effect on DNA uptake by the corresponding competent bacteria [5], [8], [9], [22], [32], [41]. Therefore, USS and DUS refer to sequence variants of the same genetic element; which makes the case for a common nomenclature. This should not be USS, given that neither USSs nor DUSs actually signal DNA uptake, as bacteria need to be competent beforehand (*i.e.*, non-competent cells will not take up DNA, no matter how many USSs or DUSs one gives them). DUS is not a satisfactory name either, as both competent Pasteurellaceae and Neisseriaceae species also take up DNA that lacks USS/DUS, though less efficiently [22], [32]–[34]. Here I suggest the unifying and more accurately descriptive name of DNA Uptake Enhancing Sequences (DUES) to highlight that both sequences are variants of the same genetic element which enhance the uptake of DNA rather than cause it.

DUESs genomic overrepresentation raises questions about their function and how the normally small bacterial genomes tolerate them in such large quantities. Among the several intracellular functions suggested [22], [23] none seems to be supported by the genomic distributions of these sequences (see Introduction) as: *(i)* DUESs are not palindromes, and most are neither inverted repeats nor biased towards locations downstream coding sequences—as it would normally have been expected from sequences terminating transcription, *(ii)* they show no orientation bias around the chromosome—feature expected from sequences interacting with the replication machinery—and we do not know of any intracellular DUES-binding protein, and *(iii)* they are not as evenly spaced as expected from sequences that help compact the chromosome. Bacterial mate recognition system, discussed in [24], is the only non-intracellular function proposed for these sequences, and the only one to account for their involvement in DNA uptake. The differences between the DUESs of the two groups of Pasteurellaceae species [16], and between these and the Neisseriaceae DUES ([5], [13]; Figure 3A;B) clearly supports this last hypothesis. Nevertheless, competence is suggested to be a mechanism more for nutritional than for sex and recombination purposes [26]–[31] and, even if this was not the case, some significantly distant bacterial species share the same DUES. These include: *(i) H. influenzae*, *P. multocida*, *H. somnus*, *A. actinomycetemcomitans* and *M. succiniciproducens*, *(ii) A. pleuropneumoniae* and *M. haemolytica* [6], [9], [13]–[16], [18] and *(iii) N. gonorrhoeae* and *N. meningitidis* [7], [10]–[13]. On the other hand, the concept of bacterial species is still a matter for debate [42]–[45] and, in some cases, including *Neisseria* [46], differentiation between species seems to be rather fuzzy. Furthermore, even if competence was for nutritional purposes, some of the DNA taken up still survives digestion and recombines with the chromosome. Occasional recombination with potentially harmful DNA from distantly related species may, thus, be a sufficient driving force for DUESs to evolve as a mechanism for minimizing such unwanted recombination. DUESs might also not be that advantageous, thus representing a sort of ‘lucky DNA’ probably driven to overrepresentation in genomic locations where they cause insignificant loss of fitness due to a possibly coincidental specificity of the protein that binds DNA (*i.e.*, receptor) at the cell surface during competence. Every DNA-binding protein has some sort of inherent specificity towards a combination of nucleotides due to its amino acids sequence and spatial configuration [47].

The possible implication of DNA uptake bias towards DUESs on the accumulation of these sequences in the genome was oddly overlooked. Earlier we [15], hypothesized that the biased DNA receptor at the cell surface of a competent species may gradually enrich its genome with DNA fragments containing the preferred DNA sequence (*i.e.*, DUES) and some of its mutated forms. Mutations towards the preferred DUES in the DNA of the ‘donor’ cell may preferentially spread to other cells, by uptake and recombination, when the ‘donor’ cell dies. On the other hand, mutations away from the DUES in the recipient chromosomes may be restored by uptake and recombination with incoming DNA from ‘donors’ with less-mutated sequences. This way, the combined action of mutation, uptake bias and reduced constraints in some genomic regions could be sufficient for DUESs to gradually accumulate in susceptible parts the genome and no additional function is needed. This would explain DUESs preferential location in non-coding regions [15], and in parts of the coding regions where they have little influence on protein configuration/function [23].

If such interpretation is correct, DNA uptake should leave a footprint on the genome, in form of higher overrepresentation of sequences depending on their similarity to the DUES, which is exactly what this work shows. Sequence overrepresentation is observed even for some mutated DUESs, where it negatively correlates to the mismatch load, and DUES nucleotides seem to emerge and start accumulating as soon as the third match. Simulations of DUES evolution in hypothetical genomes subjected to mutation and uptake bias towards the DUES only fail to reproduce the observed distribution of sequence overrepresentation across mismatch loads, whilst consideration of additional uptake bias towards mismatched sequences did. This, together with the clear correlation between genomic overrepresentation and uptake efficiency, suggests that mutations and DNA uptake bias result in a force sufficient enough to allow DUES gradual evolution by accumulation of point mutations in susceptible parts of the genome (*i.e.*, by molecular drive). This is supported by the experimentally detectable uptake of DNAs from unrelated species (*i.e.*, with mismatched or no DUESs) by competent Pasteurellaceae and Neisseriaceae species [22], [32]–[34].

The higher overrepresentation of *N. gonorrhoeae* DUES compared to that of *H. influenzae* could be due to the almost permanent character of competence in the first species [36], [37] as opposed to its occasional nature in the latter [4], [38], [39]. Even so, the increase in sequence overrepresentation accompanying the decrease in mismatch load is not linear in any of the two species, as we see sharp drops between the DUES and its singly-mismatched forms and, to a lower degree, between the latter and sequences with two mismatches. This distribution could reflect a strong specificity of the DNA-binding receptor, as well as the length of the evolutionary history of the DNA uptake bias and/or its efficiency. In principle, the more specific the receptor is, the more pronounced the overrepresentation of the DUES compared to its mismatched forms will be. Similarly, the longer the uptake bias was running for, or the more efficient it is, the closer genomes will be to an equilibrium, where the relative overrepresentations of the DUES and its mismatched forms better reflect the differences in the relative efficiency of their uptake.

In spite of all the similarities in genomic frequencies and evolutionary dynamics between *H. influenzae* and *N. gonorrhoeae* DUESs, their actual sequence consensuses are different both in shape and composition. In this work I identify less conserved nucleotides previously unreported at each side of the *N. gonorrhoeae* DUES core, bringing the consensus up to 16 bp, with four additional nucleotides at the 5′ side of the core (two of which strongly conserved) and two at the 3′ side. The resulting consensus is identical to that of *N. meningitidis* DUES reported in [13], suggesting common ancestry of the DUESs of these two sister species. Therefore, just like the Pasteurellaceae [15], *Neisseria* species seem to have ancestral DUES-mediated preferential uptake of conspecific DNA. The obvious reason for the differences between *H. influenzae* and *N. gonorrhoeae* DUESs is differences in structure and binding specificity of the DNA receptors at the cell surfaces of both species. PilC is the extracellular 110 kDa adhesin [48] suggested as binding DNA at the tips of *N. gonorrhoeae*'s type IV pili [49], [50]. Its equivalent at the tips of *H. influenzae* type IV pili are a 216 amino acids protein (called HifD) and two units of a 435 amino acids protein (called HifE) [51]–[55]. Given this difference, it is hard to refrain from speculating that whilst PilC could be the receptor driving the entire *N. gonorrhoeae* DUES, HifD might be binding to and driving the core sequence of *H. influenzae* DUES, whereas the two HifE proteins could have specificity towards the two almost identical less conserved regions of this DUES. However, this can only be confirmed after proper experimental testing (*e.g.*, gene knockouts, DNA-protein cross-linking, southern-blotting, band-shifts and DNaseI foot-printing, antibodies…).

The results of this work also show that, at least for *H. influenzae*, only one DUES seems to be bound by the receptor at the cell surface at any given time, independently of its orientation or position in the DNA fragment. A similar result was experimentally obtained in *A. actinomycetemcomitans* [9], suggesting that this is a general characteristic of DUES mediated DNA uptake. In addition, the most similar sequence to the DUES in a DNA fragment seems to be the one most likely to be bound. Both characteristics are of importance to the molecular drive model of DUES evolution postulated in [15], since the simultaneous binding of more than one DUES would not explain the genomic distribution of these sequences—it favors significant clustering in some chromosomal regions. In addition, bias towards DUESs in a particular orientation would have caused bias in their orientation around the chromosome. Indiscriminate binding of the receptor to sequences independently of their degree of similarity to the DUES, however, would loosen or even abolish the drive responsible for the emergence and gradual accumulation of DUESs in the genome.

Several genomically overrepresented oligonucleotides, both pro- and eukaryotic, seem to be selectively driven in a similar mode as the DUES, whilst those underrepresented seem to be gradually eliminated (dragged) in an inverse fashion. This suggests that the gradual mode of sequence evolution discussed above might be a general feature of short DNAs selectively driven or dragged to genomic over- or underrepresentation, including protein/transcription factor-binding, palindromic, repeated and other oligonucleotides. Statistical deviation from the expected frequencies [56]–[59] is used as indicator of DNA functionality, including transcription-factor binding [60]–[62]. DUESs themselves resemble sequence families that interact with sequence-specific DNA-binding proteins (*e.g.*, CRP, LexA and other pro- and eukaryotic transcription factor-binding DNAs); which typically consist of a short conserved core extended by less conserved regions clustering around a consensus sequence without exactly reproducing it (see [63]–[65]).

In conclusion, the results of this work suggest a gradual accumulation of genomic oligonucleotides by molecular drive towards overrepresentation due to the combined effects of mutation, the function of the oligonucleotide and some of its mutated forms, and weak selection against changes in the functionally unconstrained regions of the genome. Underrepresented oligonucleotides, however, seem to be gradually eliminated by a molecular drag emanating from the combined effects of mutation, the negative effect of the oligonucleotide and some of its mutated forms on the fitness of the carrier, and selection against changes in the functionally constrained regions of the genome. Several oligonucleotides seem to evolve in this way, including transcription factor/protein-binding and functionally constrained palindromic and repeated DNAs. DUESs themselves might be a sort of ‘lucky DNA’ driven to genomic overrepresentation, in the susceptible parts of the genome, not due to any other function but the possibly coincidental specificity of the receptor that binds DNA for uptake during natural competence for transformation.

## Materials and Methods

### Theoretical analysis

#### Sequence frequencies and overrepresentation

The programs Sequence_Extractor.pl (a modified version of the Perl based program search_USSmissmatches_sequence.pl, see acknowledgements) and BioEdit version 7.0.1 were used to search both strands of the genomes of H. influenzae str. Rd KW20 (Pasteurellaceae, 1.83 Mb genome size, 38.1% G+C, Accession no. NC_000907) and N. gonorrhoeae str. FA1090 (Neisseriaceae, 2.154 Mb genome size, 51.5% G+C, Accession no. NC_002946) and count the number of their respective DUES and all its mutated forms. The same was done for three control sequences (CS) with the same base composition but no match to the DUES. These were CSH1 = 5′-GGTCAGTAG, CSH2 = 5′-TGCGAGATG and CSH3 = 5′-CGAGTGTAG for H. influenzae, and CSN1 = 5′-ATTCAGCCGG, CSN2 = 5′-TAATGGCCCG and CSN3 = 5′-AGGAGTCTCC for N. gonorrhoeae. In addition, similar analysis was carried out for H. influenzae and N. gonorrhoeae DUESs in the genomes of Streptococcus pyogenes str. M1 GAS (Streptococcaceae, 1.85 Mb genome size, 38.5% G+C, Accession no. NC_002737) and Chlorobium tepidum str. TLS (Chlorobiaceae, 2.155 Mb genome size, 56.5% G+C, Accession no. NC_002932), which have no DUES-biased DNA uptake and their genomes are of similar sizes and G+C contents as those of H. influenzae and N. gonorrhoeae.

The expected number of sequences at each mismatch load was calculated as: 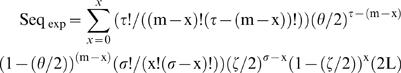
(equation 1), where *m* is the mismatch load analyzed, τ is the number of adenines and thymines (Ws) in the DUES, σ is the number of cytosines and guanines (Ss) in the same DUES, *x* is the maximum number of mismatches that could affect DUES W positions at the mismatch load *m*, θ is the genomic frequency of Ws, ς is the genomic frequency of Ss and *L* is the genome's length in base pairs. Sequence representation was calculated at each mismatch load as *Rep_Seq_* = (*Seq_obs_*−*Seq_exp_*)/*Seq_exp_* (equation 2), where *Seq_obs_* is the observed number of sequences. Significant deviation of the observed numbers of sequences from the expected ones was tested using the goodness of fit Chi-squared (χ*^2^*), which values were multiplied by |*Seq_obs_*−*Seq_exp_*|/(*Seq_obs_*−*Seq_exp_*) (equation 3) to differentiate overrepresentation from underrepresentation.

#### Computer simulations

To test the contribution of DNA uptake bias to the observed distribution of sequence frequencies, I wrote the Perl based program Genome_Dynamics.pl (program available on request). It simulates the evolution of the genomic frequencies of a given sequence and all its mismatched forms starting from a given distribution until the equilibrium or any other target. Each generation, a mismatch load looses the sequences mutated to the immediately higher and lower mismatch loads while gaining some of the sequences mutating from the same. Assuming there is no other evolutionary force than single (i.e., point) mutation, sequences at a mismatch load m should decay (i.e., mutate to mismatch load m+1) at the proportion μ(*Seq*)(1−(*m*/*n*)) (equation 4) per mutation per generation, and improve (i.e., mutate to mismatch load m-1) at μ(*Seq*)(*m*/(*3n*)) (equation 5), where μ is the mutation rate per base per generation, Seq is the number of sequences at the mismatch load and generation analysed, and n is the size of the sequence in bp. Uptake biases U_0_ to U_n_ in favour of sequences at the mismatch loads 0 to n should result in additional generational increase of the frequency of sequences at any mismatch load m by the proportion U_m_ of sequences mutated from the mismatch loads m−1 and m+1. In addition, there will be a generational decrease by the proportion U_m−1_ of sequences improving from the mismatch load m to m−1, and the proportion U_m+1_ of sequences decaying from the load m to m+1—note that U_m_ could also include other selective forces than DNA uptake. Repetition of these calculations at each mismatch load for many generations allows simulating the evolution of a given initial distribution of sequence frequencies in a population under a given mutation rate and distribution of uptake bias values towards sequences at different mismatch loads. Genome_Dynamics.pl assumes panmixia and I used a population of 10^18^ individuals (i.e., cells) evolving at 10^−2^ mutation per base per generation.

#### DNA Sequence logos and DUES nucleotides evolution

DNA sequence logos were generated in order to detect possible nucleotide conservation outside the 9 or 10 bp DUES, and to graphically monitor the emergence and evolution of DUES nucleotides in the respective genomes. I used the program Sequences_Extractor.pl to search both strands of H. influenzae and N. gonorrhoeae genomes and extract all the 100 bp sequences containing the respective DUES or any of its mismatched forms at position 38 from the 5′ end of the sequence. I then used WebLogo version 2.8.2 [66], [67] to generate logos for the sequences extracted at each mismatch load. Deviation of the genomic G+C content from the 50% assumed by WebLogo was considered by amending its source code and adding a correction factor F to the value of each base frequency used for generating the logo. F was calculated as |*V*|(*0.5*−θ)/θ (equation 6) for Ws, and |*V*|(0.5−ζ)/ζ (equation 7) for Ss, where V is the value being corrected. This adjustment permits more accurate assessment of the selective pressure on DUES Ss and Ws by minimising underestimation of the importance of the less frequent ones.

#### Nucleotide genomic overrepresentation and sequence quantification

The genomic representation of each nucleotide at each position of the sequences at each mismatch load was calculated as *Rep_Nuc_* = (*Nuc_obs_*−*Nuc_exp_*)/*Nuc_exp_* (equation 8), where Nuc_obs_ and Nuc_exp_ are the observed and the expected numbers of the nucleotide at the position and sequences analysed.

Contrarily to the unbiased distribution of nucleotides in sequence parts not targeted by the extraction program (positions outside the DUES core), the distribution of nucleotides within DUES core positions was biased due to targeting of specific combinations of nucleotides by the program Sequences_Extractor.pl. Thus, for nucleotides in the first type of positions, Nuc_exp_ was calculated as θ*Seq_obs_*/*2* (equation 9) for Ws, and ζ*Seq_obs_*/*2* (equation 10) for Ss. Whereas for matches at DUES core positions, Nuc_exp_ was calculated using equation 11 for Ws, and 12 for Ss. For mismatches at DUES core positions, however, calculation of Nuc_exp_ depends on the nature of both the mismatched nucleotide in question and the DUES match at the same position. Thus, for a W as mismatch at a DUES core position, Nuc_exp_ was calculated using equation 13, if the match at the same position is also a W, and 14, if it was an S. For an S as mismatch at a DUES core position, Nuc_exp_ was calculated using equation 15, if the match at the same position is a W, and 16, if it was an S as well.

Equation 11:
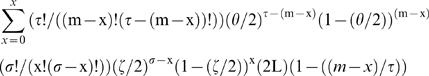
Equation 12:
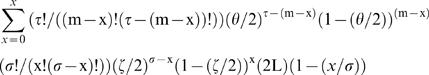
Equation 13:
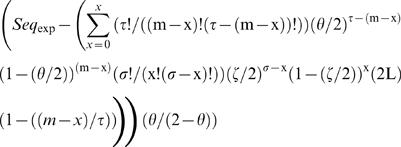
Equation 14:
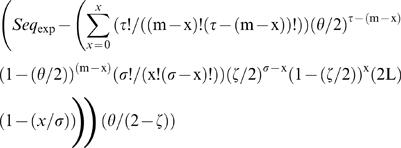
Equation 15:
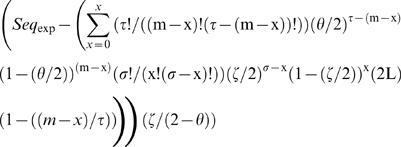
Equation 16:
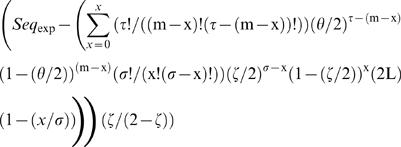
A modified version of equation 3 in [66] was used to calculate a nucleotide conservation index (Con_Nuc_) for each nucleotide at each position of sequences at each mismatch load as 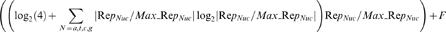 (equation 17), where Max_Rep_Nuc_ is the highest representation possible for a nucleotide at the mismatch load analyzed. *Max_Rep_Nuc_* = (*Tot_Seq_*−*Nuc_exp_*)/*Nuc_exp_* (equation 18), where Tot_Seq _is the total number of sequences at the mismatch load. This way the strength of any given sequence, of the same length (C) as the DUES consensus, can be quantified from the overall conservation of its nucleotides across mismatch loads as 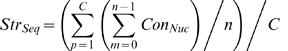 (equation 19), where p is the position.

#### Dependence of the uptake bias on the strength of the sequence

I searched the 28 DNA fragments tested for DNA uptake in [40] (Accession no. M33432 to M33459) and extracted then calculated the strength of all their 29 bp sequences using the program *Sequences_Extractor.pl* and equation 19. To test whether the genomic overrepresentation of DUES nucleotides reflects the efficiency of their uptake, I tested the dependence of the uptake of these 28 DNA fragments on the strength of their 29 bp sequences by regression analyses using *Statistica version 5.1*. As negative control the positions of these 29 bp sequences were randomized, using a script written in *Perl*, and a similar regression analysis performed on the resulting sequence strengths and the uptake of the DNA fragment from where the sequences were originally extracted.

#### Experimental analysis

To further test the theoretical results, synthetic DNA sequences containing the most conserved *H. influenzae* DUES consensus or one of its mutated forms were tested for uptake efficiency by competent *H. influenzae*. Both a 29 bp sequence of the most frequent nucleotide at each *H. influenzae* DUES consensus position (best DUES) and another of the least frequent ones (worst sequence) were synthesized and cloned by blunt-end ligation into the *Sma*I site of the plasmid pGEM-7Zf(-) (Accession no. X65311). Control 222 bp DNA template fragments containing the best DUES or the worst sequence were then PCR amplified from the constructs. Sequences carrying point mutations towards the least frequent nucleotide were generated from the best DUES construct using a three-step cohesive-end PCR process. For each desired mutant, two half-fragments (113 bp and 135 bp, with 26 bp overlap) were first produced using overlapping internal primers mutated at the chosen DUES position. After gel electrophoresis and purification, both PCR products were combined as template for a third cohesive-end PCR reaction. The final 222 bp PCR products were then gel purified and sequenced before use as templates for Klenow radio-labeling reactions. These were carried out for three hours at room temperature and, for the initial hour, contained limiting α-^33^P-dATP (6.0 µM). Subsequent addition of unlabeled (cold) dATP ensured complete replication of each molecule. DNA was then gel purified, and incorporation of α-^33^P-dATP checked by autoradiography after electrophoresis of an aliquot in acrylamide gel. Radio-labeled DNAs were then mixed with unlabelled DNAs of the same sequence to a specific radioactivity of 1,000 counts per minute (cpm) per ηg of DNA. 10, 20, 30 or 40 ηg of each DNA sequence was then separately added to 0.5 ml of competent *H. influenzae* cells for transformation as described in [68]. After 15 min incubation at 37 C in rotating tubes, 25 µl of ice cold DNaseI (1 mg/ml) was added to each tube before gentle vortexing and incubation for 5 min on ice. 50 µl of ice cold 5 M NaCl was then added and the tubes gently vortexed then centrifuged at 16,000 g for 1 min at 4 °C. After re-suspension in ice-cold MIV medium [39] containing 1 M NaCl, gentle vortexing and centrifugation, the final pellets were re-suspended in scintillation vials containing 200 µl MIV medium and 1 ml aqueous scintillation fluid at room temperature. Scintillation count was carried out in a Beckman Scintillation Counter. For standardization and accuracy, vials were counted simultaneously, P^33^ decay factor considered, and the background's P^33^ cpm subtracted. The nanograms of DNA taken up were estimated using its specific radioactivity. Experiments were carried out in triplicate.

## Supporting Information

Table S1 Goodness of fit Chi-squared test (*χ* ^2^) on the observed and expected numbers of DUESs, control sequences, as well as all their mismatched forms.
(0.12 MB DOC)
