# Supplementary material for: Genome Dynamics of Short Oligonucleotides: The Example of Bacterial DNA Uptake Enhancing Sequences
Source: PLoS One. 2007 Aug 15;2(8):e741. doi: 10.1371/journal.pone.0000741 (PMC1939737; doi:10.1371/journal.pone.0000741)
Supplement: Table S1 — Goodness of fit Chi-squared test (χ 2) on the observed and expected numbers of DUESs, control sequences, as well as all their mismatched forms. (0.12 MB DOC) [file pone.0000741.s001.doc]

| **Table S1.** Goodness of fit Chi-squared test (*χ^2^*) on the observed and expected numbers of DUESs, control sequences, as well as all their mismatched forms. | | | | | | | | | | | | | | | |
| --- | --- | --- | --- | --- | --- | --- | --- | --- | --- | --- | --- | --- | --- | --- | --- |
| Mismatch load | *H. influenzae* (1.83 Mb, 38.1% G+C) | | | | | | | | | | | | *S. pyogenes* (1.85 Mb, 38.5% G+C) | | |
|  | HDUESH | | | CSH1 | | | CSH2 | | | CSH3 | | | HDUESS | | |
|  | *Seq_exp_* | *Seq_obs_* | *Χ^2^* | *Seq_exp_* | *Seq_obs_* | *χ^2^* | *Seq_exp_* | *Seq_obs_* | *χ^2^* | *Seq_exp_* | *Seq_obs_* | *χ^2^* | *Seq_exp_* | *Seq_obs_* | *χ^2^* |
| 0 | 8 | 1471 | 267546.704 | 8 | 2 | -4.500 | 8 | 5 | -1.125 | 8 | 2 | -4.500 | 9 | 4 | -2.778 |
| 1 | 253 | 761 | 1020.084 | 253 | 96 | -97.420 | 253 | 278 | 2.470 | 253 | 113 | -77.464 | 264 | 169 | -34.183 |
| 2 | 3358 | 4097 | 162.779 | 3358 | 1751 | -768.332 | 3358 | 3455 | 2.804 | 3358 | 1956 | -584.807 | 3474 | 2795 | -132.587 |
| 3 | 25702 | 27487 | 124.838 | 25702 | 18473 | -2018.867 | 25702 | 26475 | 23.410 | 25702 | 18848 | -1814.842 | 26394 | 23891 | -235.660 |
| 4 | 124856 | 127852 | 74.420 | 124856 | 108279 | -2123.482 | 124856 | 127044 | 39.689 | 124856 | 109506 | -1820.735 | 127455 | 121831 | -239.327 |
| 5 | 399114 | 399632 | 0.752 | 399114 | 390331 | -169.652 | 399114 | 399473 | 0.361 | 399114 | 390127 | -177.627 | 405531 | 404144 | -4.159 |
| 6 | 839240 | 829358 | -81.816 | 839240 | 867857 | 1268.929 | 839240 | 836918 | -4.503 | 839240 | 872863 | 1752.465 | 849937 | 860211 | 161.283 |
| 7 | 1119274 | 1108405 | -59.193 | 1119274 | 1168064 | 3081.679 | 1119274 | 1114679 | -10.544 | 1119274 | 1158639 | 2003.686 | 1131376 | 1143006 | 172.329 |
| 8 | 859177 | 862651 | 18.343 | 859177 | 851952 | -42.133 | 859177 | 859723 | 0.451 | 859177 | 846644 | -126.932 | 867978 | 866528 | -1.680 |
| 9 | 289291 | 298566 | 322.918 | 289291 | 253475 | -4057.557 | 289291 | 292230 | 32.411 | 289291 | 261582 | -2428.015 | 292466 | 282307 | -322.713 |
| Mismatch load | *N. gonorrhoeae* (2.154 Mb, 51.5% G+C) | | | | | | | | | | | | *C. tepidum* (2.155 Mb, 56.5% G+C) | | |
|  | NDUESN | | | CSN1 | | | CSN2 | | | CSN3 | | | NDUESC | | |
|  | *Seq_exp_* | *Seq_obs_* | *Χ^2^* | *Seq_exp_* | *Seq_obs_* | *χ^2^* | *Seq_exp_* | *Seq_obs_* | *χ^2^* | *Seq_exp_* | *Seq_obs_* | *χ^2^* | *Seq_exp_* | *Seq_obs_* | *χ^2^* |
| 0 | 5 | 1965 | 768320.889 | 5 | 1 | -3.200 | 5 | 0 | -5.000 | 5 | 0 | -5.000 | 5 | 4 | -0.200 |
| 1 | 134 | 774 | 3056.811 | 134 | 245 | 91.950 | 134 | 61 | -39.767 | 134 | 8 | -118.474 | 145 | 138 | -0.338 |
| 2 | 1788 | 2954 | 760.652 | 1788 | 4769 | 4972.062 | 1788 | 1055 | -300.371 | 1788 | 455 | -993.371 | 1930 | 1949 | 0.187 |
| 3 | 14134 | 19001 | 1681.453 | 14134 | 21238 | 3582.360 | 14134 | 11110 | -644.859 | 14134 | 6392 | -4226.791 | 15148 | 15420 | 4.901 |
| 4 | 73297 | 87214 | 2688.309 | 73297 | 96158 | 7254.298 | 73297 | 67419 | -463.226 | 73297 | 50535 | -6946.889 | 77754 | 78654 | 10.608 |
| 5 | 260499 | 282572 | 1991.330 | 260499 | 286144 | 2688.130 | 260499 | 259944 | -1.106 | 260499 | 235547 | -2237.113 | 272773 | 277025 | 70.761 |
| 6 | 642576 | 635257 | -68.766 | 642576 | 633834 | -98.116 | 642576 | 656476 | 353.752 | 642576 | 657443 | 404.496 | 662385 | 668878 | 75.223 |
| 7 | 1086293 | 1030667 | -1904.168 | 1086293 | 1015177 | -3119.616 | 1086293 | 1101764 | 294.965 | 1086293 | 1149902 | 5005.815 | 1099462 | 1096068 | -6.891 |
| 8 | 1204485 | 1160334 | -998.975 | 1204485 | 1143291 | -1925.525 | 1204485 | 1190233 | -103.456 | 1204485 | 1259795 | 3543.384 | 1193897 | 1173674 | -212.135 |
| 9 | 791002 | 812380 | 708.482 | 791002 | 821326 | 1426.197 | 791002 | 782821 | -65.612 | 791002 | 757007 | -1135.489 | 765930 | 763196 | -7.650 |
| 10 | 233632 | 274730 | 7648.246 | 233632 | 285665 | 12261.499 | 233632 | 236965 | 50.273 | 233632 | 190764 | -7419.227 | 220463 | 234887 | 994.752 |
| *Seq_exp_*.: Expected number of sequences, *Seq_obs_*: Observed number of sequences. Sequence names as in Figure 1, and significant *χ^2^* values are underlined. Note that positive *χ^2^* indicates overrepresentation, whereas a negative one indicates underrepresentation (equation 3). | | | | | | | | | | | | | | | |
